# Inverse Immunological Responses Induced by Allergic Rhinitis and Head and Neck Squamous Cell Carcinoma

**DOI:** 10.1371/journal.pone.0086796

**Published:** 2014-01-22

**Authors:** Camilla Rydberg Millrud, Terese Hylander, Susanna Kumlien Georen, Åsa Kågedal, Ola Winqvist, Lars Olaf Cardell

**Affiliations:** 1 Division of ENT diseases, Department of Clinical Science, Intervention and Technology, Karolinska Institutet, Karolinska University Hospital, Stockholm, Sweden; 2 Department of Medicine, Translational Immunology Unit, Karolinska Institutet, Stockholm, Sweden; Beijing Institiute of Otolaryngology, China

## Abstract

Several epidemiological studies have investigated the relation between allergy and cancer with contradicting conclusions, and reports on immunological differences are scarce. By focusing on inflammation, the present study was designed to compare the immune response induced by allergic rhinitis (AR) and head and neck squamous cell carcinoma (HNSCC). Blood and serum was obtained from patients with symptomatic seasonal AR, and newly detected HNSCC, as well as healthy controls. Peripheral blood mononuclear cells (PBMC) and polymorphonuclear leukocytes (PMN) were isolated and cultured with or without the toll-like receptor ligands, Pam_3_CSK_4_, LPS, R837, and CpG. Cellular activation and cytokine release were assessed with ELISA, Luminex Multiplex Immunoassay, flow cytometry, and real-time RT-PCR. Sera from HNSCC patients showed elevated levels of innate immune cytokines, and exhibited a response profile consistent with an increased innate immune reaction. In contrast, sera and stimulated PBMC from AR patients displayed increased concentrations of T cell related cytokines, consistent with an adaptive immune response. The presented data demonstrate that AR and HNSCC induce two distinct immunological processes, indicating an inverse association between the immunological responses seen in patients with allergy and cancer of the upper airway.

## Introduction

The existence of a possible association between allergic airway inflammation and cancer has been debated over the past several years. Because reports on immunological differences are scarce, the discussion has almost entirely been based on information derived from epidemiological studies with diverse outcomes [1], [2], [3], [4], [5].

Allergic inflammation is a lymphocyte mediated disease with a T helper (Th)2 dominated phenotype [6]. The adaptive immune system, especially T cells, is important in cancer [7]. Patients with head and neck squamous cell carcinoma (HNSCC) have been demonstrated to have a shift towards the pro-tumorigenic Th2 response that inhibits the anti-tumor activity of the Th1 reaction [8], [9]. The innate immune system is interposed as a bridge between the external environment and the adaptive system [10]. Pattern-recognition receptors (PRRs), especially toll-like receptors (TLRs), are known to have an important role in this interplay [11]. The present study focus on differences in the immunological reactions seen during ongoing allergic rhinitis (AR) and early stages of still untreated HNSCC, with focus on effects induced by external TLR stimulation. The HNSCC investigated were found foremost in the oral and nasopharyngeal regions, and the allergic individuals were sampled during pollen season. Cells were stimulated with the TLR agonists Pam_3_CSK_4_ (TLR1/2), LPS (TLR4), R837 (TLR7) and CpG (TLR9). Subsequently, the cell activation was analyzed for CD11b which is involved in leukocyte recruitment, the interleukin-2 (IL-2) receptor CD25 presented on activated T cells, CD69 a very early activation marker, and CD98 an early marker for T cell activation. In addition, the cytokine profile was investigated, and IL-6 and IL-8, secreted by activated innate immune cells, were analyzed.

## Materials and Methods

### Ethics Statement

This study was approved by the Ethics Committees of Karolinska Institutet and Lund University, and a written consent was obtained from all participants.

### Patients

The study included 13 patients (8 females and 5 males) with symptomatic, pollen-induced AR and 10 patients (3 females and 7 males) newly diagnosed with HNSCC prior to initiation of treatment, along with 18 healthy controls (15 females and 4 males). The median age of the allergic patients was 42 years (range 25–77), HNSCC patients 67 years (range 51–80), and controls 52.5 years (range 26–69). Allergic individuals that exhibited a positive skin prick test towards birch and/or grass were sampled during pollen season, when their disease was the most active. At the time of sampling, the patients had to fill in a symptom score to verify that they had an ongoing allergic reaction. They also had to avoid allergy medications a week before blood samples were collected. The HNSCC patients were sampled in connection with the detection of their disease, prior to initiation of treatment. None of the cancer patients were on immune modulating medication, and they had no previous history of malignant disease. The control individuals were healthy volunteers without medication. The tumor (T) and lymph node (N) classification of the cancer patients at the time of inclusion are shown in Table 1. The study included cancer patients with the following tumor localization: 7 oral, 2 nasopharyngeal, and 1 glottic.

**Table 1:** Clinical tumor (T) and lymph node (N) classification of the head and neck squamous cell carcinoma (HNSCC) patients.

N stage		0	1	2	3	*Total*
**T stage**	**1**	1	1	1	0	*3*
	**2**	2	0	1	0	*3*
	**3**	1	0	3	0	*4*
	**4**	0	0	0	0	*0*
*Total*		*4*	*1*	*5*	*0*	*10*

### Cell Line

The human pharyngeal squamous cell carcinoma cell line FaDu (HTB-43; ATCC; Manassa, VA) was cultured in minimum essential medium (MEM) with Earl’s salts and 2 mM L-glutamine (GIBCO, Grand Island, NY), and supplemented with 10% FBS (PAN Biotech, Aidenbach, Germany), and 100 U/ml penicillin and 100 µg/ml streptomycin (GIBCO). Cells were cultured in tissue culture flasks at 37°C in a humidified 5% CO_2_ air atmosphere.

### Cell Separation

Freshly drawn blood was diluted 1∶1 with PBS and centrifuged using Ficoll-Paque (Amersham Bioscience, Uppsala, Sweden). The interphase containing peripheral blood mononuclear cells (PBMC) was collected and washed in PBS before resuspended in complete medium, consisting of RPMI-1640 supplemented with 0.3 g/l L-glutamine (Sigma Aldrich, St. Louis, MO), 100 U/ml penicillin and 100 µg/ml streptomycin (GIBCO), and 10% autologous plasma, to a concentration of 1×10^6^ cells/ml. The polymorphonuclear leukocytes (PMN) rich pellet was recovered and treated with ammonium chloride erythrocyte lysis solution (0.8% NH_4_Cl, 10 mM KHCO_3_, and 0.1 mM EDTA) for 15 min on ice. The cell suspension was centrifuged at 400 g, washed in PBS, and resuspended in complete medium to a density of 4×10^6^ cells/ml.

### Cell Culture

Both PBMC and PMN were treated with 1 µg/ml Pam_3_CSK_4_, 1 µg/ml LPS, 5 µg/ml R837 (Invivogen, San Diego, CA, USA), 0.3 µM CpG (sequence 5′-tcgtcgttttgtcgttttgtcgtt-3′; DNA technology, Aarhus, Denmark), or with vehicle (as the control). PBMC was cultured for 24 or 72 h, and PMN for 4 or 24 h at 37°C in a humidified air atmosphere with 5% CO_2_. Thereafter, the supernatants were analyzed with ELISA, Luminex Multiplex Immunoassay, and the cells with flow cytometry. PMN obtained from healthy individuals was also cultured in the presence or absence of culture medium supernatants from FaDu, and stimulated with or without 1 µg/ml LPS for 4 h at 37°C in a humidified air atmosphere with 5% CO_2_. Subsequently, the supernatant were analyzed for cytokine secretion with ELISA.

### Elisa and Luminex Multiplex Immunoassay

The levels of IL-6 (Limit of detection (LOD): 2 pg/ml) and IL-8 (2 pg/ml) in cell culture supernatants from PMN were analyzed with ELISA plates from eBioscience (San Diego, CA, USA). In addition, levels of 17 cytokines were measured in supernatants from PBMC using Bio-Plex Pro Human Cytokine 17-plex Assay from Bio-Rad Laboratories (Herculeas, CA, USA) according to the manufacturer’s instructions. The following cytokines were measured: IL-1β (0.6 pg/ml), IL-2 (1.6 pg/ml), IL-4 (0.7 pg/ml), IL-5 (0.6 pg/ml), IL-6 (2.6 pg/ml), IL-7 (1.1 pg/ml), IL-8 (1.0 pg/ml), IL-10 (0.3 pg/ml), IL-12p70 (3.5 pg/ml), IL-13 (0.7 pg/ml), IL-17 (3.3 pg/ml), granulocyte-colony stimulating factor (G-CSF; 1.7 pg/ml), granulocyte monocyte-colony stimulating factor (GM-CSF; 2.2 pg/ml), interferon (IFN)-γ (6.4 pg/ml), monocyte chemotactic protein (MCP)-1 (1.1 pg/ml), macrophage inflammatory protein (MIP)-1β (2.4 pg/ml), and TNF-α (6.0 pg/ml). The cytokine levels were measured by the Bio-Plex 200 system (Bio-Rad).

### Antibodies

The following antibodies (Abs) were purchased from BD Bioscience (San Jose, CA, USA): CD4-APC (clone: RPA-T4), CD11b-APC (ICRF44), CD16-FITC (3G8), CD25-FITC (M-A251), CD62L-PE (DREG-56), CD69-APC-Cy7 (FN50), and CD98-FITC (UM7F8).

### Flow Cytometry

Flow cytometry analyses were performed on a Beckman Coulter FC500 (Marseille, France) or a BD LSRFortessa flow cytometer (BD Bioscience). Events in the range 50 000–100 000 were collected depending on the occurrence of the investigated leukocyte population. Data was analyzed using the FlowJo software (Tree Star Inc., Ashland, OR, USA). To ensure flow cytometric standardization, the voltage settings were updated daily using FlowSet calibration beads. All Abs were titrated before use. For staining, cells were incubated with Abs for 20 min, washed and resuspended in PBS prior to analysis.

### Real-Time RT-PCR

PBMC was lysed in RLT buffer (Qiagen, Hilden, Germany) supplemented with 1% 2-mercaptoethanol and stored in −80° until use. RNA was extracted using the RNeasy Mini Kit (Qiagen) according to manufacturer’s instruction. The RNA quality and quantity was determined by spectrophotometry, based on the A_260_/A_280_ ratio (1.7–2.1 in all samples). Reverse transcription of RNA into cDNA was carried out using the Omniscript Reverse Transcriptase Kit (Qiagen) and Oligo(dT) primer (DNA Technology) in a Mastercycler personal PCR machine (Eppendorf AG, Hamburg, Germany). Real-time PCR was performed in a Stratagen Mx3000P (Agilent Technologies Inc., Santa Clara, CA) using Brilliant II SYBR® Green QPCR Master Mix (Agilent Technology) to detect COX-1 (PTGS1; Hs00924808_ml) and COX-2 (PTGS2; Hs00153133_ml). The thermal cycler was programmed as followed: initial set up for 10 min at 95°C, 45 cycles of denaturation at 95°C for 15 s each and thereafter annealing/extension at 60°C for 1 min. The gene expression was assessed using the comparative threshold cycle (C_t_) method [12].

### Statistics

Statistical analyses were performed using GraphPad Prism 5 (GraphPad Software, San Diego, CA, USA), and data are presented as mean ± standard error of the mean (SEM). *n* is equal to the number of independent donors, and a *p*-value of ≤0.05 were considered statistically significant. The nonparametric Mann-Whitney test was used to determine the statistical difference between the groups, and for paired data the nonparametric Wilcoxon signed rank test was used.

## Results

### Distinct Serum Cytokine Profiles in Patients with Allergy and Head and Neck Cancer

**Figure 1:**
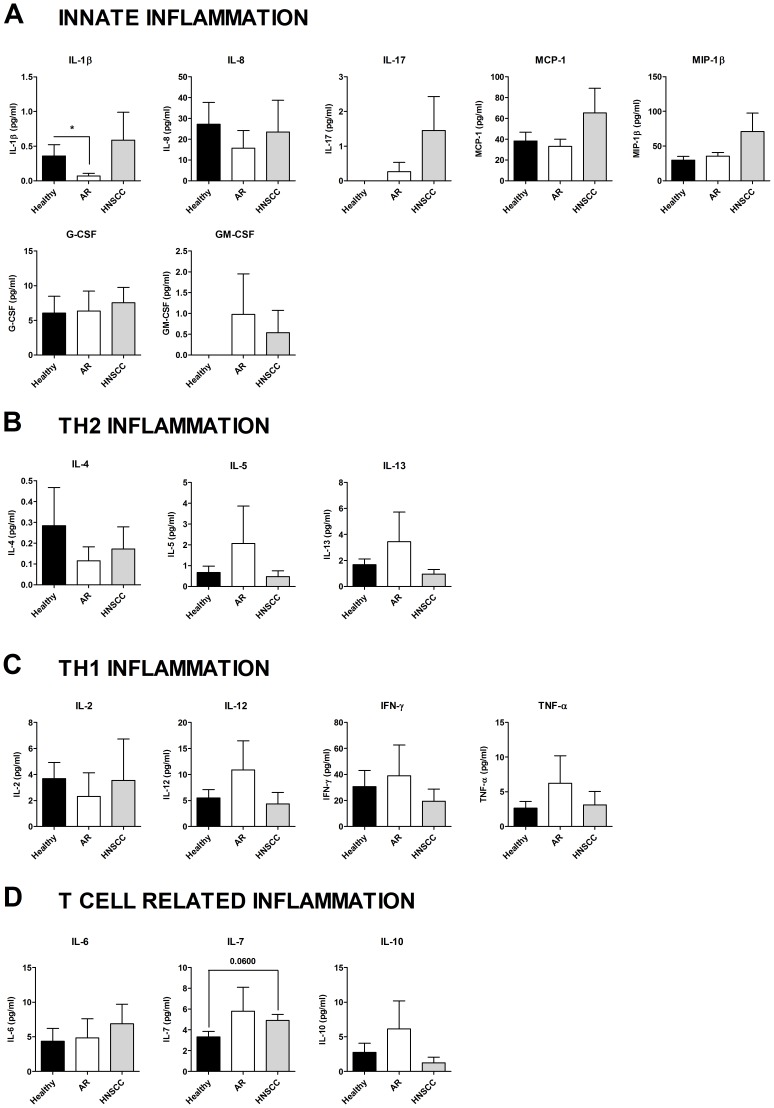
Cytokine concentrations in serum. Sera were collected from healthy controls (n = 10), patients with an ongoing seasonal allergic rhinitis (AR; n = 13) and patients with head and neck squamous cell carcinoma (HNSCC; n = 8), and analyzed for cytokine levels with Luminex Multiplex Immunoassay. *p≤0.05.

Cytokine levels in serum samples from healthy controls, AR patients, and HNSCC patients were analyzed with Luminex Multiplex Immunoassay. Cytokines involved in innate inflammation, IL-1β, IL-17, MCP-1, MIP-1β, and G-CSF, all showed a suggestive tendency to be increased in HNSCC patients as compared to AR patients. GM-CSF was the only exception. However, the concentration of IL-1β and IL-17 were close to the LOD value 0.6 pg/ml for IL-1β, and 3.3 pg/ml for IL-17 (Figure 1A). In addition, IL-7 was increased, and IL-6 was slightly elevated in sera from HNSCC patients compared to healthy controls (Figure 1D). As expected, sera from allergic individuals displayed an increased level of the Th2 cytokines IL-5 and IL-13 compared to sera from healthy controls and HNSCC patients (Figure 1B). Only very low levels close to detection limit of IL-4 were found (Figure 1B). In addition, sera from AR patients displayed a significant decrease in the serum levels of the innate cytokine IL-1β compared to sera from healthy controls (Figure 1A). More surprisingly, the serum levels of the Th1 cytokines IL-12, IFN-γ, and TNF-α were increased in AR patients compared to sera from healthy controls and patients with HNSCC patients (Figure 1C).

### PMN from Head and Neck Cancer Patients Respond with Increased Activation after TLR Stimuli

**Figure 2:**
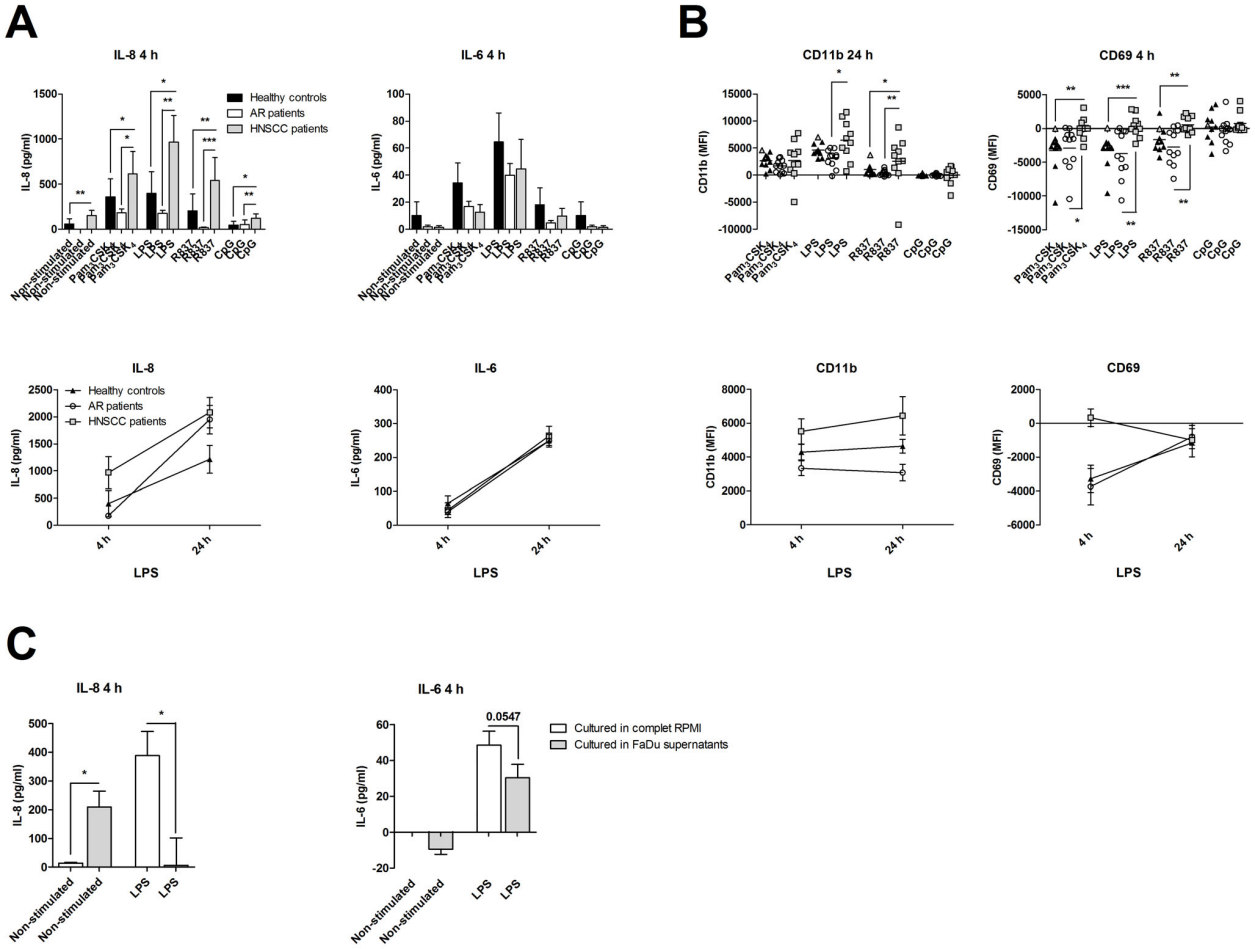
Increased activation of polymorphonuclear leukocytes (PMN) in patients with head and neck squamous cell carcinoma (HNSCC). Blood was obtained from healthy controls (n = 10), patients with an ongoing seasonal allergic rhinitis (AR; n = 11) and patients with HNSCC (ELISA n = 9; FACS n = 10). PMN were isolated and cultured in the presence or absence of Pam_3_CSK_4_ (1 µg/ml), LPS (1 µg/ml), R837 (5 µg/ml) or CpG (0.3 µM) for 4 and 24 h. (**A**) The cell free supernatants were then analyzed for IL-6 and IL-8 with ELISA, (**B**) and the CD16 positive cells were investigated for the expression of CD11b and CD69 using flow cytometry. The results are presented as the non-stimulated value minus the TLR stimulated values. Grey colored samples were analyzed on a BD LSRFortessa, whereas the rest of the samples were investigated on a Beckman Coulter Navios flow cytometer. (**C**) PMN from healthy individuals (n = 9) were incubated with or without culture medium supernatant from the HNSCC cell line FaDu, and stimulated with or without 1 µg/ml LPS for 4 h, and analyzed for IL-6 and IL-8 secretion with ELISA. The basal cytokine levels in the media were subtracted from the concentrations present in the PMN cultures. MFI = mean fluorescence intensity; *p≤0.05; **p≤0.01; ***p≤0.001.

To determine the differences in leukocyte functionality between patients with AR, HNSCC, and healthy controls, PMN were isolated and stimulated with or without TLR ligands (Pam_3_CSK_4,_ LPS, R837, and CpG) for 4 and 24 h, and subsequently analyzed with ELISA and flow cytometry. PMN obtained from HNSCC patients displayed an increased basal secretion of IL-8, and exhibited a higher IL-8 response to the different TLR ligands compare to the response of PMN from AR patients and healthy controls after 4 h (Figure 2A). When investigating the 24 h time point, the IL-8 production was equal in the supernatant from AR and HNSCC patients when compared to the control. In contrast, the IL-6 release remained the same in all three groups (Figure 2A and Figure S1A). When studying the expression of cell surface markers, we found significantly increased expression levels (mean fluorescent intensity; MFI) of CD11b and the early activation marker CD69 on stimulated PMN isolated from the HNSCC patients compared to PMN from AR patients and healthy controls (Figure 2B). The increased expression levels of CD11b was found on foremost LPS stimulated PMN after both 4 and 24 h, whereas the elevated level of CD69 was observed for all types of stimuli except CpG after 4 h of stimulation (Figure 2B and Figure S1B, C).

To investigate whether it is the tumor that dictates the activation pattern of PMN, PMN from healthy donors were cultured in supernatants from the HNSCC cell line FaDu with or without LPS for 4 h, using IL-8 and IL-6 secretion as read outs. Since the FaDu supernatants contained a basal level of IL-8 and IL-6, the concentration of these cytokines in the media alone was subtracted from PMN cultured samples. The IL-8 levels were decreased in LPS stimulated cells in FaDu supernatant. The unstimulated PMN controls cultured in the presence of FaDu supernatants were found to have a decreased level of IL-6 expression compared to the FaDu medium alone and the unstimulated PMN in complete RPMI medium. LPS stimulated cells cultured in FaDu supernatants were also found to release a lower amount of IL-6 compared to LPS treated PMN in complete RPMI medium (Figure 2C).

### Decreased Activation Of PBMC from Head and Neck Cancer Patients after TLR Stimuli

**Figure 3:**
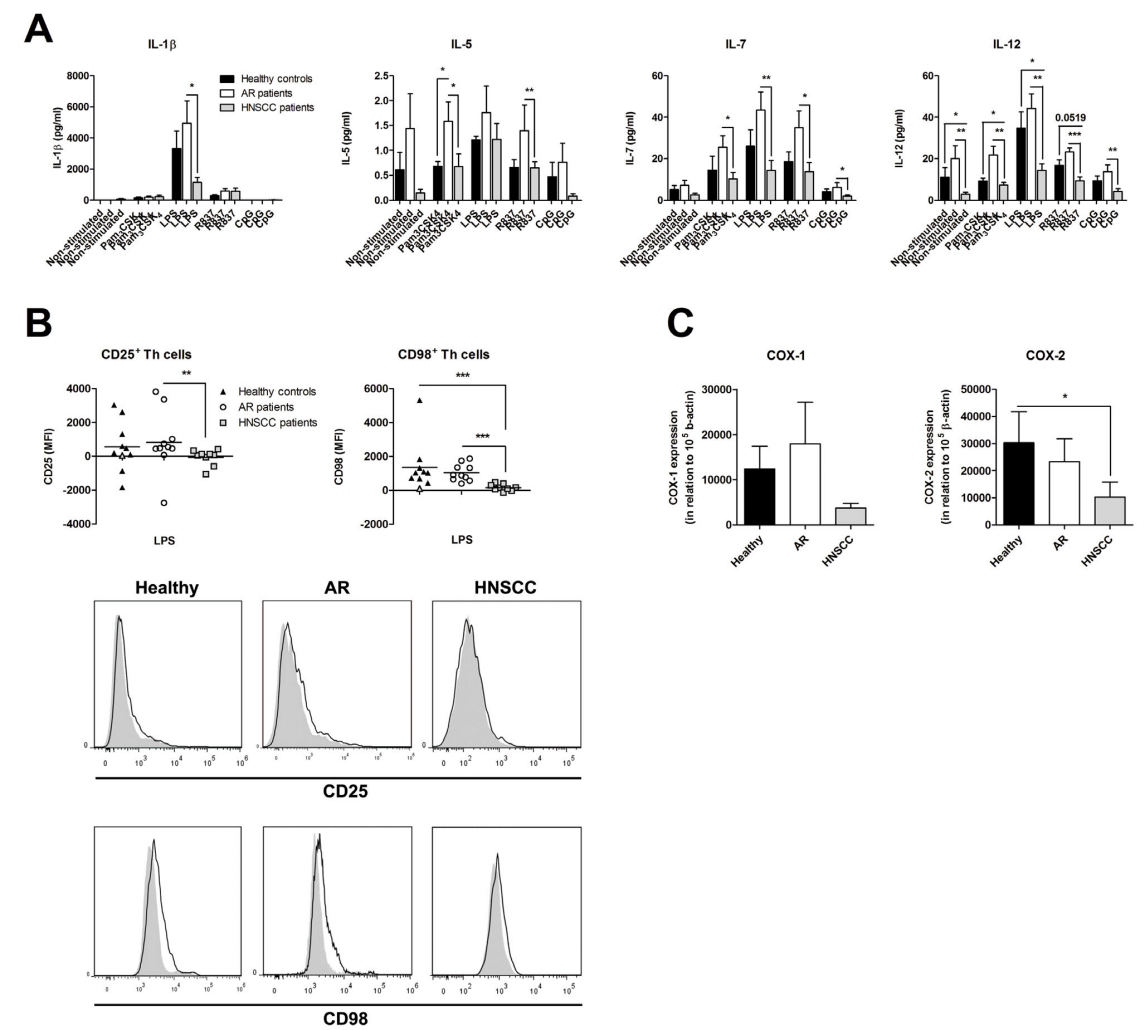
Decreased peripheral blood mononuclear cells (PBMC) activation in patients with head and neck squamous cell carcinoma (HNSCC). PBMC were isolated from blood collected from healthy controls (n = 10), patients with an ongoing seasonal allergic rhinitis (AR; n = 11) and patients with HNSCC (n = 9), and cultured with or without Pam_3_CSK_4_ (1 µg/ml), LPS (1 µg/ml), R837 (5 µg/ml) or CpG (0.3 µM) for 24 h. (**A**) Thereafter, the supernatants were analyzed for the secreted cytokine profile with Luminex Multiplex Immunoassay, 4 out of 17 investigated cytokines are shown, (**B**) and the cells were examined for the expression of CD25 and CD98 on CD4 positive T helper (Th) cells with flow cytometry. The results are presented as the non-stimulated value minus the TLR stimulated values. Grey colored samples were analyzed on a BD LSRFortessa, whereas the rest of the samples were investigated on a Beckman Coulter Navios flow cytometer. One representative histogram from the healthy controls, AR patients and HNSCC patients is displayed. The open histograms represent LPS stimulated cells, and the light grey histograms are denoting the non-stimulated samples. (**C**) RNA was extracted from peripheral PBMC, isolated from healthy individuals (n = 8), AR patients (n = 10), and HNSCC patients (n = 7), made into cDNA, and thereafter analyzed for COX-1 and COX-2 mRNA expression with real-time PCR. Data are given in relation to the housekeeping gene β-actin, 100 000×2^−ΔCt^, and presented as mean ± SEM. MFI = mean fluorescence intensity; *p≤0.05; **p≤0.01; ***p≤0.001.

Further, the functionality of PBMC from healthy controls, AR individuals and HNSCC patients was determined. PBMC were isolated, stimulated with or without Pam_3_CSK_4,_ LPS R837, or CpG for 24 and 72 h. Cytokine profiles were analyzed with Luminex Multiplex Immunoassay, and T cell activation was monitored by flow cytometry. Four representative cytokines are displayed in Figure 3 and the other 13 cytokines can be found in Figure S2. PBMC obtained from the cancer patients showed a decreased IL-1β secretion after 24 h of LPS stimulation compared to PBMC from the AR individuals. Low levels of IL-5, IL-7 and IL-12 were also released from PBMC isolated from HNSCC patients, whereas PBMC from AR patients showed high concentrations of these cytokines (Figure 3A). No differences in cytokine levels were observed between 24 and 72 h. The HNSCC patients also displayed a decreased Th cell activation as observed by a reduction in MFI of CD25 and CD98 after both 24 (Figure 3B) and 72 h (Figure S3A, B). However, a high percentage of CD98-positive Th cells were also found in the cancer patients (Figure S3B). Finally, real-time RT-PCR was used to determine the expression of COX-1 and COX-2 mRNA in PBMC obtained from healthy individuals, AR patients and HNSCC patients. PBMC isolated from cancer patients were found to display a lower expression of COX-2 compared to PBMC from healthy controls (Figure 3C).

## Discussion

This study demonstrates that allergy and cancer induce two distinct immunological reactions. The tumorigenic immune response is dominated by an innate immune reaction, and by suppressed T cells, whereas the allergic reaction is characterized by an adaptive response with enhanced activation of T cells.

The innate and neutrophil dominated inflammation seen among the HNSCC patients is characterized by a strong PMN activation, and a suggestive tendency of high serum levels of IL-1β, IL-17, MCP-1, MIP-1β, and G-CSF. These findings are well in line with previous studies reporting altered peripheral PMN activation in HNSCC [13], [14]. The presented data show that PMN isolated from patients with HNSCC have an increased basal secretion of IL-8 that was confirmed to be mediated by the tumor. This highlights that the tumor cells and its release of mediators modulate the activation process of PMN. The suppressed release of IL-8 from LPS stimulated PMN from healthy donors cultured in FaDu supernatants indicates that the tumor cells release mediators that inhibit the TLR4 signaling pathway for IL-8 secretion. These inhibitory substances seem to be effective locally in the tumor without reaching the circulation, since PMN isolated from the blood of patients with HNSCC displayed an increased IL-8 release after TLR stimulation. This is in concert with previous observations of tumor cells expressing and releasing mediators that modulate the immune system in its favor [15]. IL-6 is known to facilitate the proliferation and differentiation of T cells [16], [17]. The failure to induce IL-6 secretion in tumor derived PMN, and the suppressed IL-6 release from LPS stimulated PMN from healthy controls cultured in supernatants from FaDu cancer cells may therefore be a tumor induced immune evasive mechanism to attenuate the effective anti-tumoral T cell responses. This assumption is partially supported by the reduced response of T cells from HNSCC patients, and the low expression of COX mRNA levels in PBMC. The high number of CD98-positive Th cells indicates that these cells have the ability to detect unfamiliar substances. However, the low CD98 expression might indicate a deficiency in the clonal T cell expansion [18], and that the suppressed and anergic state render them incapable of eliciting a response. This might explain the increased number of activated T cells previously reported [19]. An altered T cell function has been described among HNSCC patients [20], [21]. The increased frequency of regulatory T cells in patients with HNSCC, and their suppressive function on effector T cells [22], [23] further verifies that T cells in HNSCC are generally suppressed.

In addition to its important role in the adaptive immunity, CD98 have been shown to be expressed by solid tumors where it promotes transformation, tumor growth and progression [18], [24], [25]. Center and Ginsberg conclude that the benefits of the adaptive immunity come with a price, in this case increased susceptibility to invasive cancer [18].

The high cytokine secretion by PBMC from allergic patients, the indicative tendency of increased levels of T cells related cytokines, and the suggestive reduced serum concentration of IL-1β in AR patients acknowledge the awareness of allergy as a lymphocyte mediated immune disease with relatively low innate immune reactivity detected. The findings of both Th1 and Th2 cytokines in PBMC supernatants and suggestive in sera supports recent notions that allergy is a more complex immunological disease than merely an imbalance between Th1 and Th2 activity [26].

T cells and the adaptive branch of the immune system play an important role in the defense against malignantly transformed cells, and enhanced lymphocyte activity can prevent cancer development [27], [28]. In contrast, cancer patients exhibit increased number of regulatory T cells causing a general suppression of the immune reaction. The reversed phenomenon is seen in allergic patients with a more depressed function of their regulatory T cells [29], [30] that may facilitate a more active adaptive immune response. In regard of this, we can only speculate whether the increased adaptive response in the AR patients might have an increased ability to raise a tumor specific immune response against malignantly transformed cells, thus providing protection against development of HNSCC.

To summarize, the presented results reflect the principal inflammatory mechanisms involved in allergy and head and neck cancer. The differences in the inflammatory response between AR and HNSCC are distinct despite the somewhat widespread distribution of the tumors and disease progression. The occurrence of two distinct immunological processes, in combination with results from previous epidemiological studies, suggests an inverse association between the immunological responses in patients with allergy and HNSCC.

## Supporting Information

Figure S1 **Increased activation of polymorphonuclear leukocytes (PMN) in patients with head and neck squamous cell carcinoma (HNSCC).** Blood was obtained from healthy controls (n = 10), patients with an ongoing seasonal allergic rhinitis (AR; n = 11) and patients with HNSCC (ELISA n = 9; FACS n = 10). PMN was isolated and cultured in the presence or absence of Pam_3_CSK_4_ (1 µg/ml), LPS (1 µg/ml), R837 (5 µg/ml) or CpG (0.3 µm) for 4 and 24 h. **(A)** The cell free supernatants were then analyzed for IL-6 and IL-8 with ELISA, and the CD16 positive cells were investigated for the expression of **(B)** CD11b and **(C)** CD69 with flow cytometry. The flow cytometry results are presented as the non-stimulated value minus the TLR stimulated values. Grey colored samples were analyzed on a BD LSRFortessa, whereas the rest of the samples were investigated on a Beckman Coulter Navios flow cytometer. MFI = mean fluorescence intensity; *p≤0.05; **p≤0.01; ***p≤0.001.
(TIF)

Figure S2 **Increased cytokine secretion by peripheral blood mononuclear cells (PBMC) from patients with allergic rhinitis (AR).** PBMC was isolated from blood collected from healthy controls (n = 9), patients with an ongoing seasonal AR (n = 11) and patients with head and neck squamous cell carcinoma (HNSCC; n = 9), and cultured in the presence or absence of Pam_3_CSK_4_ (1 µg/ml), LPS (1 µg/ml), R837 (5 µg/ml) or CpG (0.3 µm) for 24 h. Thereafter, the supernatants were analyzed for the secreted cytokine profile with Luminex Multiplex Immunoassay. MFI = mean fluorescence intensity; *p≤0.05; **p≤0.01; ***p≤0.001.
(TIF)

Figure S3 **Increased T helper (Th) cell activation in patients with allergic rhinitis (AR).** Peripheral blood mononuclear cells (PBMC) were isolated from blood obtained from healthy controls (24 h and 72 h n = 10), patients with an ongoing seasonal AR (24 h n = 10; 72 h n = 11) and patients with head and neck squamous cell carcinoma (HNSCC; 24 h n = 9, 72 h n = 10), and incubated with or without Pam_3_CSK_4_ (1 µg/ml), LPS (1 µg/ml), R837 (5 µg/ml) or CpG (0.3 µm) for 24 and 72 h. Subsequently, the cells were examined for the expression of **(A)** CD25 and **(B)** CD98 on CD4 positive Th cells with flow cytometry. The results are presented as the non-stimulated value minus the TLR stimulated values. Grey colored samples were analyzed on a BD LSRFortessa, whereas the rest of the samples were investigated on a Beckman Coulter Navios flow cytometer. MFI = mean fluorescence intensity; *p≤0.05; **p≤0.01; ***p≤0.001.
(TIF)
