# Supplementary figures and images for: Inverse Immunological Responses Induced by Allergic Rhinitis and Head and Neck Squamous Cell Carcinoma
Source: PLoS One. 2014 Jan 22;9(1):e86796. doi: 10.1371/journal.pone.0086796 (PMC3899344; doi:10.1371/journal.pone.0086796)

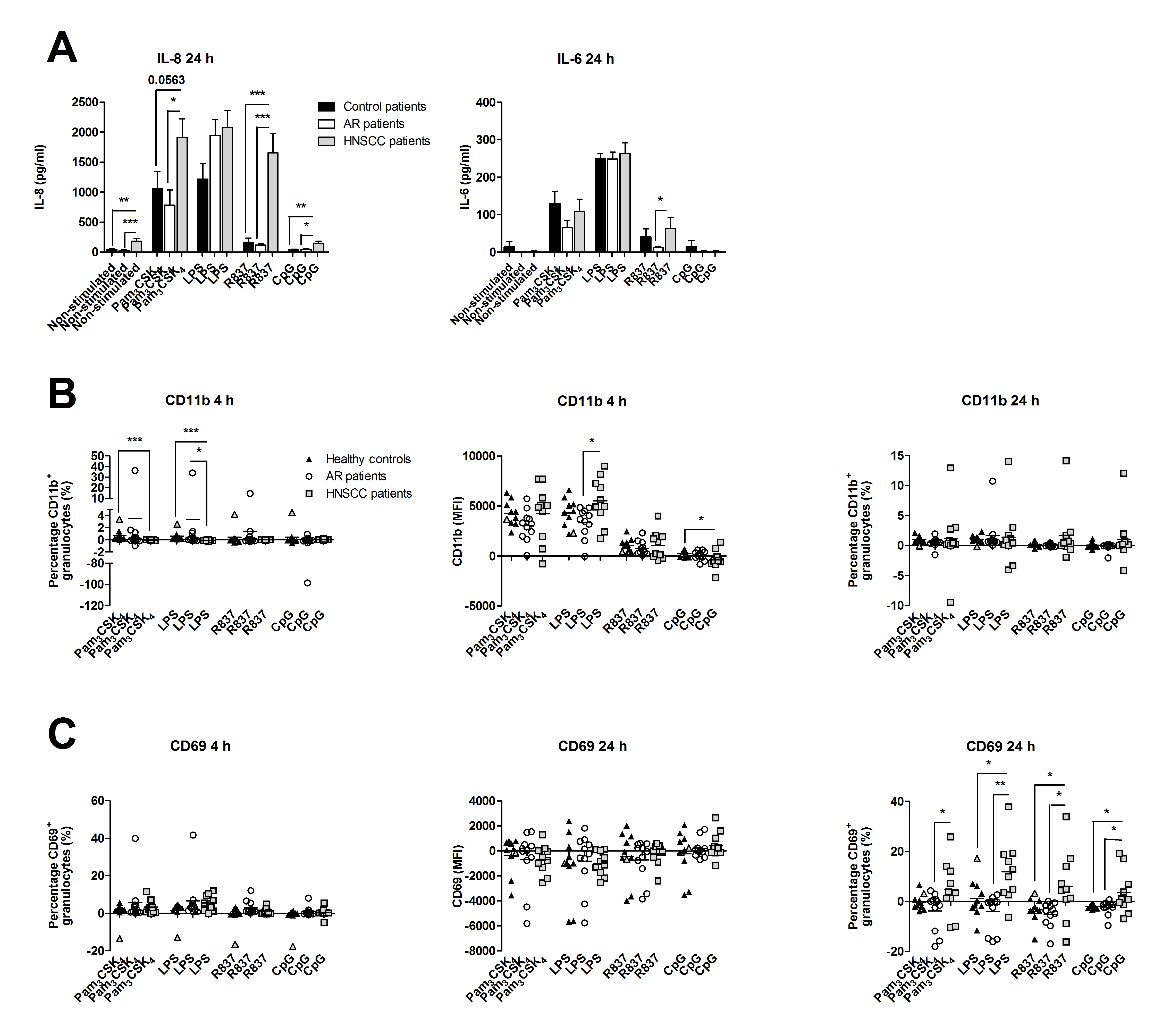

Supplement: Figure S1 — Increased activation of polymorphonuclear leukocytes (PMN) in patients with head and neck squamous cell carcinoma (HNSCC). Blood was obtained from healthy controls (n = 10), patients with an ongoing seasonal allergic rhinitis (AR; n = 11) and patients with HNSCC (ELISA n = 9; FACS n = 10). PMN was isolated and cultured in the presence or absence of Pam3CSK4 (1 µg/ml), LPS (1 µg/ml), R837 (5 µg/ml) or CpG (0.3 µm) for 4 and 24 h. (A) The cell free supernatants were then analyzed for IL-6 and IL-8 with ELISA, and the CD16 positive cells were investigated for the expression of (B) CD11b and (C) CD69 with flow cytometry. The flow cytometry results are presented as the non-stimulated value minus the TLR stimulated values. Grey colored samples were analyzed on a BD LSRFortessa, whereas the rest of the samples were investigated on a Beckman Coulter Navios flow cytometer. MFI = mean fluorescence intensity; *p≤0.05; **p≤0.01; ***p≤0.001. (TIF) [file pone.0086796.s001.tif]

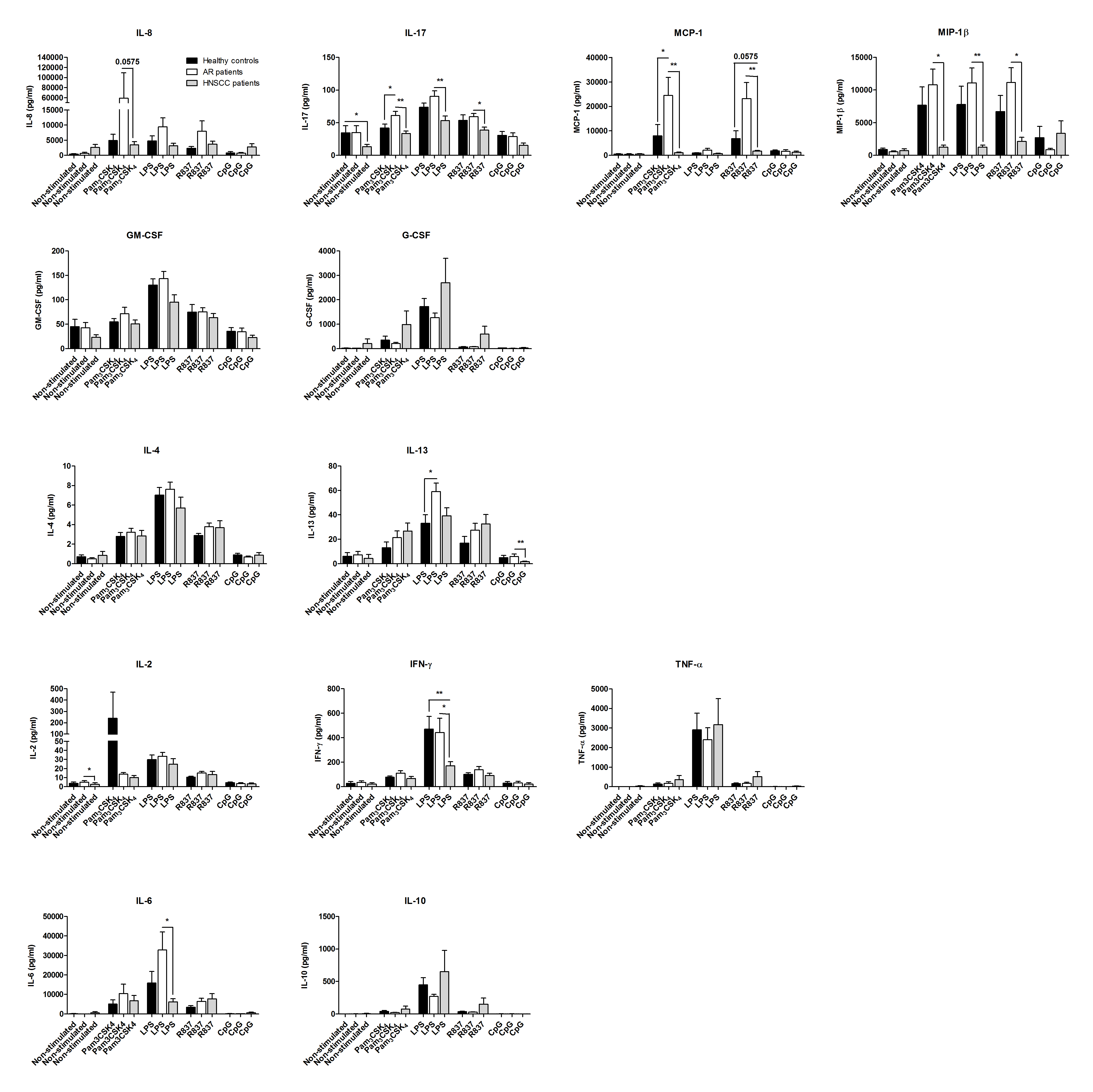

Supplement: Figure S2 — Increased cytokine secretion by peripheral blood mononuclear cells (PBMC) from patients with allergic rhinitis (AR). PBMC was isolated from blood collected from healthy controls (n = 9), patients with an ongoing seasonal AR (n = 11) and patients with head and neck squamous cell carcinoma (HNSCC; n = 9), and cultured in the presence or absence of Pam3CSK4 (1 µg/ml), LPS (1 µg/ml), R837 (5 µg/ml) or CpG (0.3 µm) for 24 h. Thereafter, the supernatants were analyzed for the secreted cytokine profile with Luminex Multiplex Immunoassay. MFI = mean fluorescence intensity; *p≤0.05; **p≤0.01; ***p≤0.001. (TIF) [file pone.0086796.s002.tif]

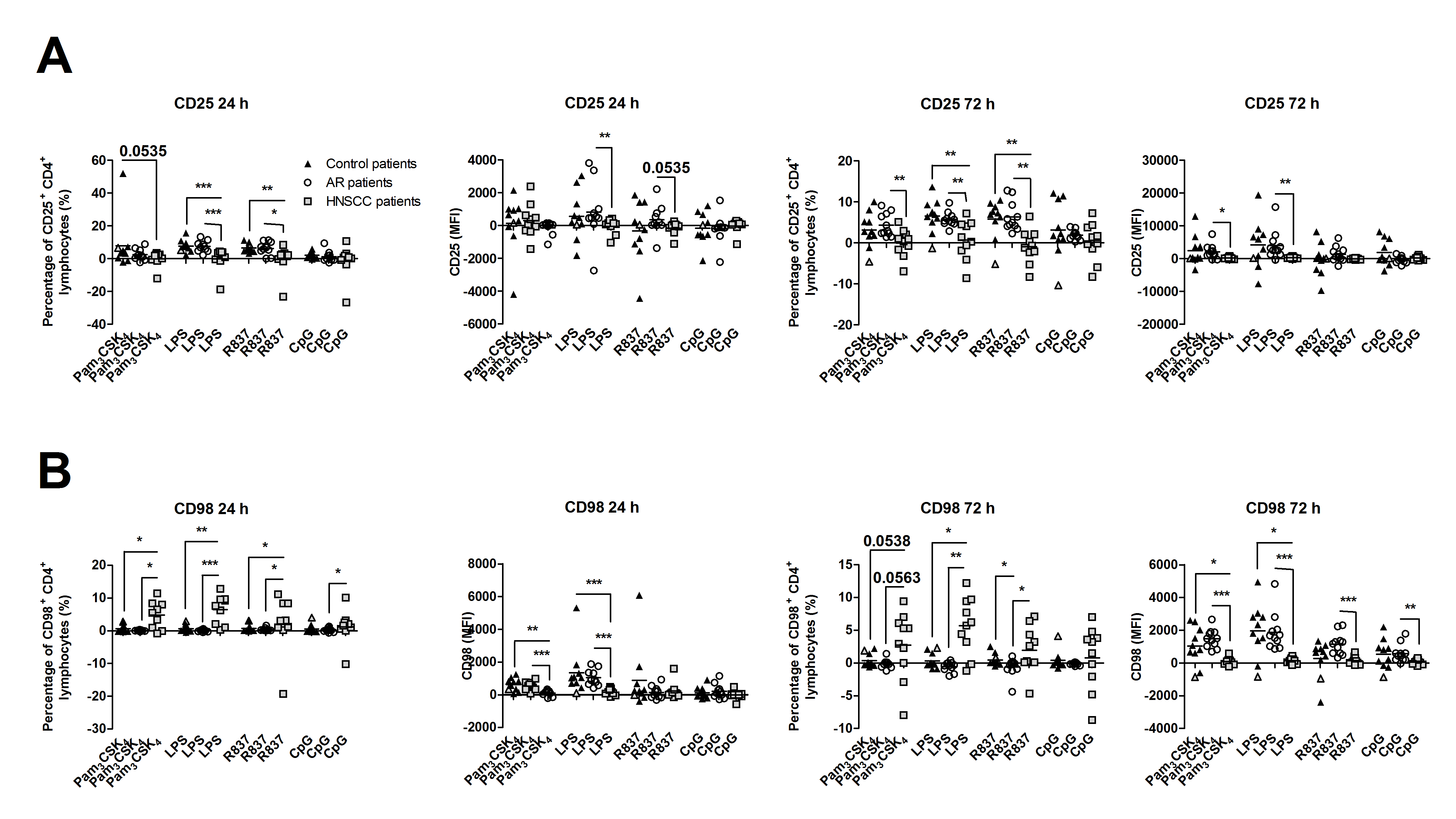

Supplement: Figure S3 — Increased T helper (Th) cell activation in patients with allergic rhinitis (AR). Peripheral blood mononuclear cells (PBMC) were isolated from blood obtained from healthy controls (24 h and 72 h n = 10), patients with an ongoing seasonal AR (24 h n = 10; 72 h n = 11) and patients with head and neck squamous cell carcinoma (HNSCC; 24 h n = 9, 72 h n = 10), and incubated with or without Pam3CSK4 (1 µg/ml), LPS (1 µg/ml), R837 (5 µg/ml) or CpG (0.3 µm) for 24 and 72 h. Subsequently, the cells were examined for the expression of (A) CD25 and (B) CD98 on CD4 positive Th cells with flow cytometry. The results are presented as the non-stimulated value minus the TLR stimulated values. Grey colored samples were analyzed on a BD LSRFortessa, whereas the rest of the samples were investigated on a Beckman Coulter Navios flow cytometer. MFI = mean fluorescence intensity; *p≤0.05; **p≤0.01; ***p≤0.001. (TIF) [file pone.0086796.s003.tif]
